# Exploring spatial and temporal patterns of visceral leishmaniasis in endemic areas of Bangladesh

**DOI:** 10.1186/s41182-017-0069-2

**Published:** 2017-11-15

**Authors:** Ashraf Dewan, Abu Yousuf Md Abdullah, Md Rakibul Islam Shogib, Razimul Karim, Md Masudur Rahman

**Affiliations:** 1 Department of Spatial Sciences, Curtin University, Perth, Australia; 2 Maternal and Child Health Division, International Centre for Diarrhoeal Disease Research, Bangladesh (icddr,b), 68 Shahid Tajuddin Ahmed Sarani, Mohakhali, Dhaka, 1212 Bangladesh; 3 Department of Geography, South Dakota State University, South Dakota, USA; 4 Center for Environmental and Geographic Information Services (CEGIS), House: 06, Road No: 23/C, Dhaka, 1212 Bangladesh

**Keywords:** Visceral leishmaniasis, Spatial heterogeneity, Geographic information system, Bangladesh

## Abstract

**Background:**

Visceral leishmaniasis is a considerable public health burden on the Indian subcontinent. The disease is highly endemic in the north-central part of Bangladesh, affecting the poorest and most marginalized communities. Despite the fact that visceral leishmaniasis (VL) results in mortality, severe morbidity, and socioeconomic stress in the region, the spatiotemporal dynamics of the disease have largely remained unexplored, especially in Bangladesh.

**Methods:**

Monthly VL cases between 2010 and 2014, obtained from subdistrict hospitals, were studied in this work. Both global and local spatial autocorrelation techniques were used to identify spatial heterogeneity of the disease. In addition, a spatial scan test was used to identify statistically significant space-time clusters in endemic locations of Bangladesh.

**Results:**

Global and local spatial autocorrelation indicated that the distribution of VL was spatially autocorrelated, exhibiting both contiguous and relocation-type of diffusion; however, the former was the main type of VL spread in the study area. The spatial scan test revealed that the disease had ten times higher incidence rate within the clusters than in non-cluster zones. Both tests identified clusters in the same geographic areas, despite the differences in their algorithm and cluster detection approach.

**Conclusion:**

The cluster maps, generated in this work, can be used by public health officials to prioritize areas for intervention. Additionally, initiatives to control VL can be handled more efficiently when areas of high risk of the disease are known. Because global environmental change is expected to shift the current distribution of vectors to new locations, the results of this work can help to identify potentially exposed populations so that adaptation strategies can be formulated.

## Background

Visceral leishmaniasis (VL) is a neglected tropical disease [1] that claims the lives of 20,000 to 40,000 people worldwide every year [2]. The disease is prevalent on the Indian subcontinent, largely affecting impoverished communities [3]. Estimates reveal that more than 67% of global VL cases occur in India, Bangladesh, and Nepal [4] and around 200–300 million people are at risk of developing VL in South Asia [3], with an estimated annual economic burden of US$350 million [5]. Because VL cases are the main driver for transmission in South Asia [6], knowledge of spatial heterogeneity is valuable for understanding its spatial and temporal patterns [7, 8].

Understanding the local or global spatial heterogeneity (clustering) of a disease can support a range of activities relevant to public health management, including disease surveillance [9], determining underlying spatial risk factors [10, 11] or causal mechanisms affecting the distribution of disease [12], understanding spatiotemporal dynamics [13], and disease control planning [2, 14]. Studies demonstrated that local and global clustering techniques are very useful for identifying statistically significant “hot” and “cold” spots of a disease, which can then be used to provide valuable insights about causative factors affecting its distribution [15, 16], particularly in regions where infection is already a concern. In a resource-poor health care system like Bangladesh, mapping disease clusters would be highly useful in planning effective strategies for control and elimination programs [17]. These maps would provide distinct information about the spatial patterns of a disease or its spatiotemporal dynamics [13, 18], which is crucial for public health officials to prioritize their activities at specific sites.

Despite VL being a major public health concern for the last few decades, little is known about the spatial heterogeneity of this disease in Bangladesh [3]. Based on the high rates of VL in Mymensingh district, a case-control study by Bern et al. [3] was possibly the first to reveal the household-level clustering of the disease in sections of a community (called *paras*). In a follow-up review paper, Bern et al. [19] assert that the VL incidence begins to diffuse in contiguous locations once areas within a village become saturated; as a result, clustering of the disease at both the household level and on a larger scale is a prominent feature. Although the work of Bern et al. [3] is useful for understanding the distribution of VL at the household (micro) level, it is unclear whether the degree of local clustering is equal across the study area or the risk of VL is equal to every spatial unit in the area of interest. In addition, Kothari et al. [20] demonstrated that the results obtained at the micro level are difficult to generalize for an entire study area owing to the fact that the “realized” niche for the sand fly vector is not equally distributed across the study area [21]. Because the incidence of VL in Bangladesh is mostly located in remote rural areas, especially in the areas with dilapidated infrastructure [22], geographical constraints often preclude an immediate response to a local epidemic. Therefore, understanding of the spatial heterogeneity of VL may help develop more effective health care systems for marginalized rural populations who are at greater risk of this fatal disease.

This study primarily aimed to assess the spatiotemporal pattern of VL cases in endemic locations of Bangladesh. Specifically, two research questions were tested in this work. First, is there any spatial heterogeneity or clustering of VL cases at the regional scale in Bangladesh? If so, to what degree does this clustering increase the risk and likelihood of disease occurrence relative to non-cluster areas?

## Methods

### Description of the study area

**Fig. 1:**
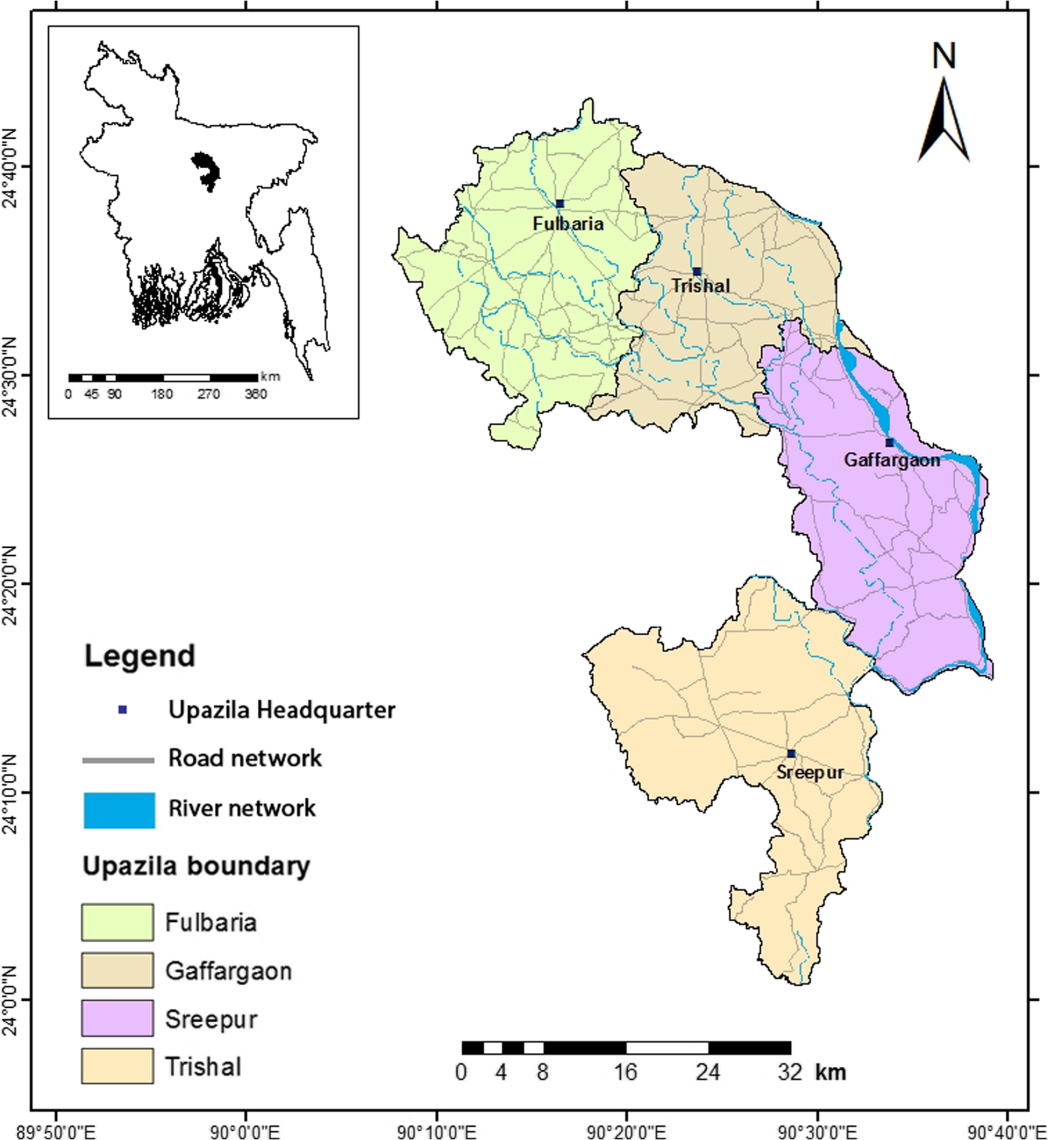
Location of the study area, showing four *upazilas* in the Mymensingh and Gazipur districts. The inset map shows the location of the study area with respect to Bangladesh

The study was conducted in four subdistricts or *upazilas* (Fulbaria, Trishal, Gaffargaon, and Sreepur) located in the hyperendemic Mymensingh district and relatively low-endemic Gazipur district [23]. The study area location is 24.19°–24.62° N latitude and 90.26°–90.54° E longitude (Fig. 1). The four *upazilas* together have a total area of 1594.93 km^2^ and population of 1,791,313, with a mean population density of 1127 people/km^2^. The average literacy rate is 46.6%, with approximately equal rates between men (47.8%) and women (45.4%). On average, 18.2% people live in pucca or semi-pucca houses, and 23.4% of the total population lack potable water. More than 52.1% of the people in the study area do not have access to safe sanitation [24].

Of the total 15,850 VL cases reported during 2008–2014 in Bangladesh, 69.12% occurred in the three *upazilas* (e.g., Fulbaria, Trishal, and Gaffargaon) in Mymensingh district and 1.8% were located in Sreepur *upazila* in Gazipur district [25]. Therefore, the study area comprises 70.92% of the nation’s total reported VL cases in preceding years, thus providing a unique opportunity to investigate spatial heterogeneity of the disease.

### Disease data

Data of reported VL cases between 2010 and 2014 were obtained from the respective *upazila* (analogous to subdistrict) health complex (UHC) in the study area. Although there is a national kala-azar treatment center, called Surya Kanta Kala-Azar Research Center (SKKRC), in Mymensingh district, we used VL cases recorded at the UHCs for a few reasons. The SKKRC hospital was established in 2012, and it started maintaining VL records from 2012 [26]. This means the use of the SKKRC database in our work would have missed a large number of VL cases from 2010 to 2011 that were recorded in the UHCs [25]. A cross-sectional study conducted on health-seeking behavior of Post-kala-azar dermal leishmaniasis (PKDL) patients in SKKRC reported that majority (88%) of the VL patients were referred from the respective UHCs who were at their second or third phase of treatment [27]. Therefore, the VL cases treated at SKKRC were very likely to have registered earlier with the respective UHCs. In addition, our database represented only the hospitalized cases, and no outpatients were included due to the fact that the outpatient data recording system lacked the multiple information checking which was however present in case of inpatient recording system [28]. Furthermore, consultation with relevant health professionals revealed that the outpatient database of a typical UHC also contains significant fake addresses. Many VL patients outside the area of interest come and register with false addresses in the UHCs of the study area, for instance, Fulbaria UHC, for availing subsidized treatments like the AmBisome (amphotericin B) treatment provided by Médecins Sans Frontières (MSF) [29].

Demographic data in the UHC log books, as well as the corresponding year and date of cases, was checked to avoid data duplication. Data of patient attributes including place of residence, date of admission/discharge, age, and sex were considered. Three out of the four *upazilas* in our study area were hyperendemic, and the UHCs received VL patients from adjacent districts and subdistricts [21, 25, 28]. We included 2082 VL cases pertaining to study area, out of 5762 cases. The excluded 3680 VL cases were from various locations and, importantly, outside from our area of interest. Inclusion of these records would have misled the cluster detection process, as respective UHC databases could not be added into our analysis, thus affecting local and global mean of VL incidences [30–32]. Finally, using a unique identifier, all VL cases were aggregated and matched with the lowest census tract polygon features (i.e., village or *mauza* shapefile), obtained from the Bangladesh Bureau of Statistics (BBS). The resultant database included annualized VL cases according to each spatial unit, from which the aggregated value for the entire study period (2010–2014) was derived by manipulating the attribute table. Likewise, population of the study area was obtained from population and housing census of 2011 [24] and linked with the appropriate geographic unit.

### Analyzing spatiotemporal patterns

To examine spatial and temporal patterns of VL occurrence in the study area, both local and global clustering techniques [33] were used. The spatial autocorrelation and the extent of global clustering were evaluated using Moran’s *I* statistic. This was done separately for each year as well as for the entire study period (2010–2014). We used the first-order Queen’s case contiguity rule with Euclidian distance to conceptualize the spatial relationship and compensate for the irregular sizes and shapes of the *mauza* boundaries used in this study. The global test examines the existence of clustering (positive or negative autocorrelation) over the area being considered and whether objects with similar values are in spatial proximity to each other [32]. Moran’s *I* ranges from + 1 (positive autocorrelation) to − 1 (negative autocorrelation), and a value of 0 corresponds to spatial randomness in distribution [34]. If global clustering was found, then a local autocorrelation algorithm was applied to the database to identify statistically significant locations of hot spots among positively autocorrelated areas (*p* < 0.05) and their spatial extent.

To examine local autocorrelation for individual years and for the entire period, the local Gi* statistic [35] was used, which takes into account VL concentration values in the neighborhood of a spatial unit [33]. To determine the statistically significant location of high or low values, the local mean infection rate is compared with the global mean rate by examining each feature within the context of neighborhood features [34]. The first-order Queen’s case polygon contiguity rule was applied to define spatial adjacency relationships among features. Use of this algorithm subsequently outputted two statistics: *z* score and *p* value for each spatial unit [36], based on which hot and cold spots of a disease could be determined. A feature with a high positive *z* score denotes a statistically significant hot spot, meaning that the spatial unit under scrutiny has a high concentration of cases and is also surrounded by features (e.g., *mauza*) with high values. When a feature produces a statistically significant negative *z* score, it has a concentration of low values and is surrounded by features with low values (cold spots) [35, 36].

In addition to the global Moran’s *I* and local Gi* statistic, we also employed a spatial scan statistic to determine space-time clusters, for several reasons. The first is to examine whether the two local methods produce consistent outcomes because different spatial techniques tend to produce different spatial patterns of a geographic phenomenon, such as a disease [37]. Second, the clusters identified through this algorithm are not defined by administrative boundaries, which minimizes preselection bias [31]. Third, because of its sensitivity to local cluster detection, statistically significant and precise clusters can be efficiently identified [9, 37]. Finally, if clusters are picked up in the same location by the two local algorithms, then the results are said to be more robust and consistent [12]; this constitutes part of our objectives.

To determine space-time clusters over the study period (2010–2014), a centroid data file was first created using the *mauza* polygon shape file that contained cases, population, and coordinate data (latitude/longitude) of each spatial unit. The Poisson probability disease model embedded in SaTScan (v. 9.4) [38] was then used to detect local clusters by scanning the entire area of interest with a cylindrical window shape. In this window, the base of the scanning cylinder represents geographic space (the radius of the window varies from zero to a user-defined upper limit) whilst the height of the cylinder represents some interval in time [11]. The scan centered the cylinder at the *mauza’s* centroid and progressively increased the radius to include 50% of the total population of each *mauza*, such that large and small clusters could be detected [10]. The default setting of no geographic overlap was used; consequently, statistically significant clusters would not overlap each other. Based on the calculated log-likelihood ratio values, the primary and secondary space-time clusters were determined [33]. For statistical significance, a simulated *p* value of the clusters was obtained through Monte Carlo testing with 999 replications [38], and cluster assessment was conducted by comparing the number of reported cases within the window with the number of expected cases.

## Results

The result of global Moran’s *I* (Table 1) showed that the distribution of VL cases was spatially autocorrelated. The largest value of Moran’s *I* was 0.315 in 2010 followed by 0.182 in 2014. Statistically significant clustering (0.195, *p* < 0.001) of the disease over the entire time span (2010–2014) was also observed (Table 1). Even though the value of Moran’s *I* decreased between 2010 and 2013, a three-time increase in Moran’s *I* was observed in 2014 compared with the preceding year, 2013.

**Table 1:** Global autocorrelation of VL (2010–2014)

Year	Moran’s *I*	*z* score	*p* value	Pattern
2010	0.315	10.997	0.001*	Clustered
2011	0.141	5.687	0.001*	Clustered
2012	0.079	2.795	0.013**	Clustered
2013	0.057	2.051	0.035**	Clustered
2014	0.182	6.982	0.001*	Clustered
2010–2014	0.195	7.582	0.001*	Clustered

*p < 0.01; **p < 0.05

**Fig. 2:**
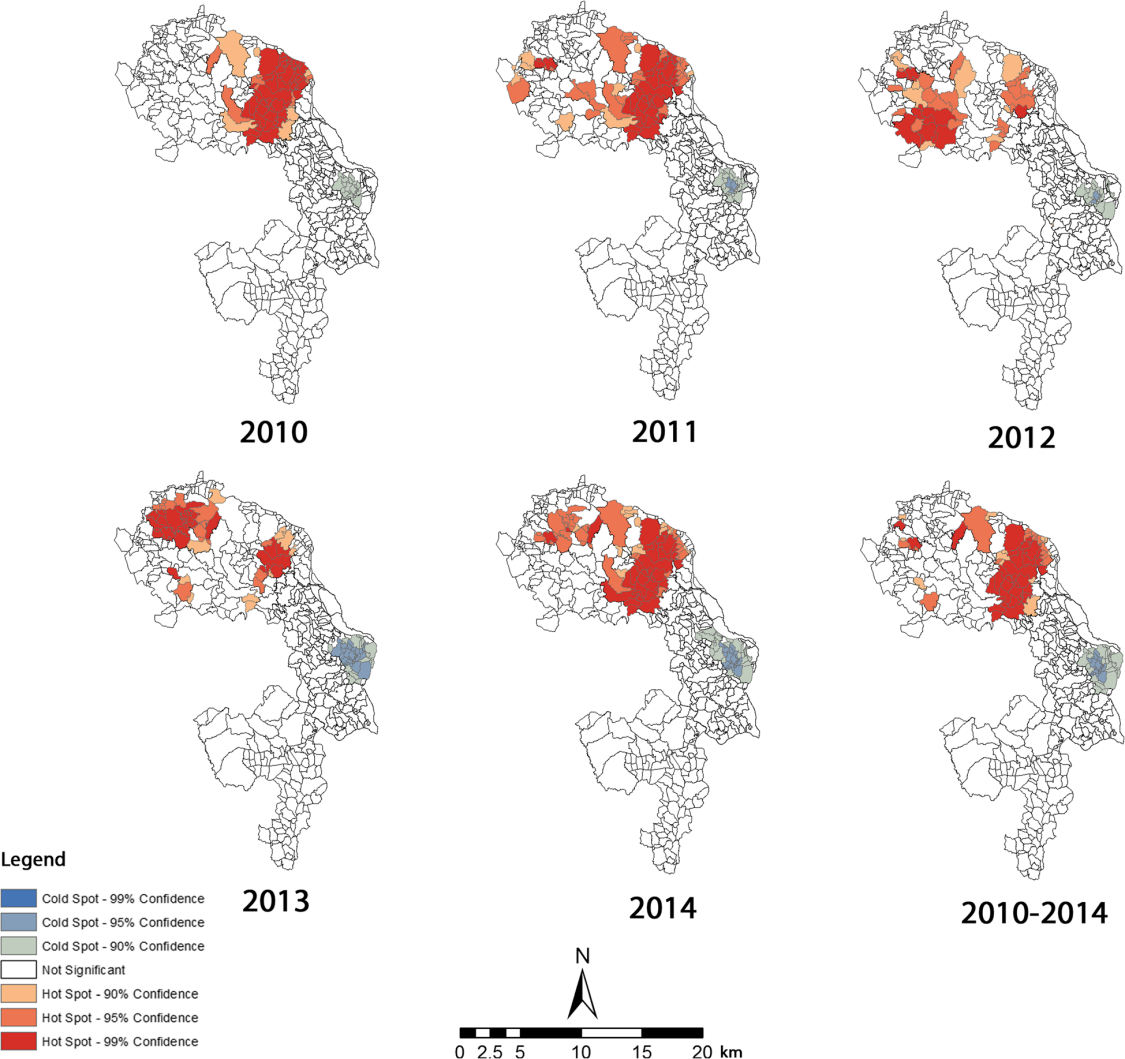
Statistically significant hot and cold spots of VL in the study area, between 2010 and 2014

Hot and cold spot analysis revealed that local clustering of VL cases varied both spatially and temporally across the study area. The spread of statistically significant (≥ 90% confidence interval) hot spots can be visualized in Fig. 2. This shows that major VL hot spots were located in Trishal *upazila* in 2010. Fulbaria *upazila* in Mymensingh district was free from VL incidence, but small pockets of clusters become visible in Fulbaria in 2011. The magnitude of the disease seemed to have reversed in 2012, with large clusters appearing in Fulbaria and few clusters in Trishal. This pattern continued in 2013; however, a pattern similar to 2011 reappeared in 2014, suggesting that Trishal had more clusters than Fulbaria, as many *mauzas* experienced higher concentrations of VL cases. Over the study period, statistically significant hot spots were found in Trishal *upazila*, with scattered small clusters in Fulbaria. The analysis suggests that the spread of VL in the study area exhibited both contiguous and relocation patterns; however, a contiguous pattern appears to be prominent, rather than the relocation type (Fig. 2). Cold spots of the disease were observed in Gaffargaon *upazila*; however, there were no hot and cold spots detected in Sreepur *upazila* of Gazipur district.

**Fig. 3:**
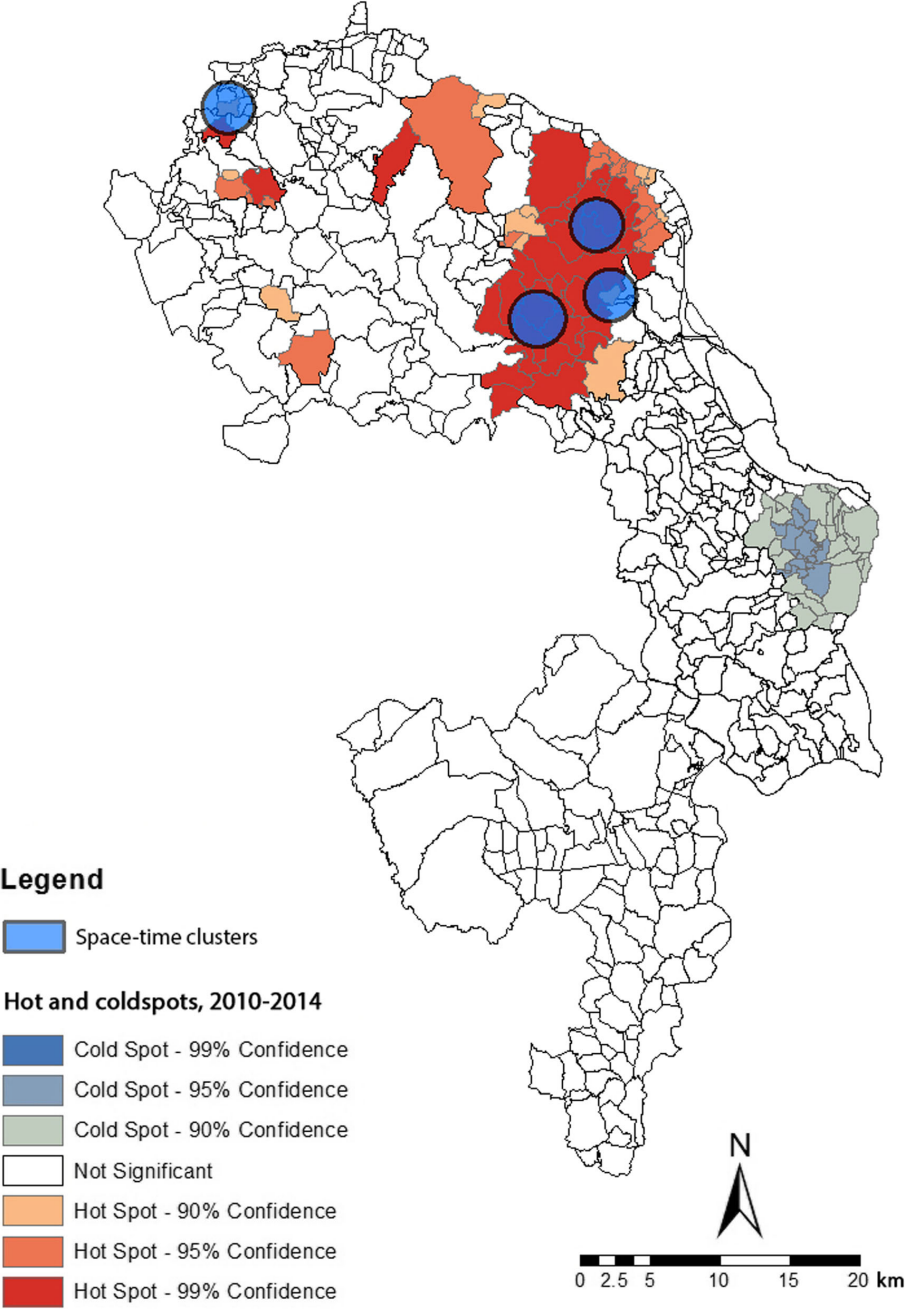
Space-time clusters (2010–2014) with respect to hot and cold spots of VL; circles represent clusters identified by SaTScan

The spatial scan test identified one primary and three secondary space-time clusters (Fig. 3), located in Trishal and Fulbaria *upazilas*. The three clusters comprised 16 *mauzas* in the study area. The most likely cluster, which included four *mauzas*, was observed in Trishal *upazila*, centered at 24.6° N, 90.5° E, with a relative risk of 11.102 (*p* < 0.001). The characteristics of the identified clusters in the study area can be found in Table 2. The log-likelihood ratio of these clusters suggests that populations within the cluster zones are at significantly greater risk of VL than those outside of clusters. Figure 3 further indicates that high-risk clusters detected by SaTScan either included or overlapped with those delineated by local Gi*.

**Table 2:** Space-time clustering of VL in the study area

	Geographic locations	Population	Reported cases	Expected cases	Relative risk	Log-likelihood ratio	*p* value	Number of locations (*mauzas)* in cluster	Number of cases in cluster	Annual cases (per 100,000)
Most likely cluster	24.60° N, 90.47° E	15,407	100	9.419	11.102	147.670	0.001	4	435	649.5
Secondary cluster 1	24.51° N, 90.43° E	8746	57	5.437	10.932	83.887	0.001	5	298	652.2
Secondary cluster 2	24.64° N, 90.21° E	5020	23	3.069	7.567	26.490	0.001	2	93	458.5
Secondary cluster 3	24.55° N, 90.47° E	13,622	32	8.328	3.887	19.540	0.001	5	96	235.1

Out of the total 1,791,313 people in the study area, 680,730 people lived within the VL-affected *mauzas*. SaTScan calculated an average annual case rate of 61 per 100,000 population within the study area. Table 2 demonstrates an annual case rate of 649.5 cases per 100,000 population within the primary cluster. This high annual case rate was followed by the three secondary clusters as well which were 652.2, 458.5, and 235.1. Hence, the primary cluster and the most significant secondary cluster demonstrated about 11 times higher annual case rate than the gross average in the study area. The observed to expected ratio for the first, second, third, and fourth clusters were 10.617, 10.660, 7.494, and 3.843, respectively. The relative risk (Table 2) also shows that the population within the clusters is significantly at higher risk of VL than the population outside the cluster.

## Discussion

Use of global and local spatial statistics revealed strong evidence of VL cases being spatially autocorrelated. Local Gi* indicated the spatial extent of hot and cold spots of VL infection in endemic areas of Bangladesh (Fig. 2). These maps may be of substantial value for determining the etiology of VL from a spatial viewpoint [39]. Even though our study did not consider how the disease dispersed in the study area, close inspection of hot spot maps (Fig. 2) obtained via local Gi* provided clues that may be used to interpret the diffusion pattern of VL over space and time. It is notable, however, that contiguous and relocation types [13, 18] of VL infection seemed to predominate throughout the study period.

Examination of individual years may provide evidence of distinct spatial pattern, which is crucial to prevent further spread of disease [18]. For instance, the 2010 hot spot map featured contiguous movement, but the disease appeared to have expanded in 2011 with single foci. It then expanded to new locations in 2012 and 2013; as a result, new hot spots emerged in areas that had no VL incidence in 2010. El-Masum et al. [40] noted a major resurgence of VL in the 1990s following the cessation of DDT (dichlorodiphenyltrichloroethane). However, a relocation type of spread to the earliest locations again became evident in 2014, and *mauzas* that were hot spots in 2013 experienced relatively few cases, resulting in the least significant hot spots (*p* < 95%). Thus, this study finding is partially in agreement with that of Bern et al. [19]. This spatial pattern may have resulted from human mobility due to work-related movement or visiting relatives. VL cases are the main driver for transmission in and around Bangladesh [6]; the role of human mobility in the spread of disease is highly likely as human migration is shown to enhance sand fly diffusion and density in areas that were previously free from infection [41]. Thus, the westward progression of hotspots during 2011–2012 and northward progression between 2012 and 2013 in Fulbaria, resulting in relocation-type diffusion of the disease, may be linked with work-related movement within the villages of the study area. Similar to our observations, Desjeux [42] identified cross-border migration as an important risk factor in transmitting VL to the Nepalese Terai region from India. In addition, ceasing of indoor residual spraying, after declines in VL incidence as reported by household members, may also account for the alternating spatiotemporal distribution of VL in the area; this however requires further investigation. Moreover, increased interaction between an ever-growing population and the natural environment in Bangladesh is providing a niche that could potentially enhance the likelihood of insurance/resurgence into new locations [21, 25]. In addition, probable ceasing of the residual spraying that had been found to be greatly effective in vector control strategies [43], with the decline of VL incidence, could account for the alternating spatiotemporal distribution of VL in the area; this however requires further investigation.

We found that both of the local clustering algorithms (e.g., Gi* and SaTScan) identified statistically significant clusters in the same locations (Fig. 3), suggesting that the results of our work are robust [12]. This means the disease did not occur at random. On closer inspection of Table 2, it can be observed that the number of reported cases was significantly higher than the number of expected cases within the clusters. This is important because the distribution of VL has not been spatially analyzed prior to the intervention design, similar to most interventions in Bangladesh [44]. However, our findings suggest that the actual nature of the disease can be heavily influenced by both spatial and temporal aspects, and areas within a cluster are exposed to greater risk than those outside of the clusters. Therefore, for most intervention programs that are simply based on the total number of reported cases, there is a risk of overlooking areas with persistent severity of disease. Out of four statistically significant clusters identified by the spatial scan test, three were located in Trishal *upazila*, a persistent VL hot spot (Fig. 3) and a very impoverished subdistrict in the study area.

Space-time analysis added additional features of VL occurrence that could otherwise be missed (e.g., only with global and local autocorrelation analyses). For example, the SaTScan analysis showed the rate of annual VL cases inside the two most significant detected clusters were 11 times higher than the average rate in the study area. Similar to our result, Singh et al. [45] reported that VL incidence in Bihar showed focal distribution with respect to space and time and found high prevalence rates within these foci [45]. Figure 3 suggests that within endemic locations, having very high VL case rates, the spatial patterns of a disease may be quite different from a space-time pattern due to the variations in incidence rate and relative risk over time. That is to say, not all the spatial clusters (2010–2014) from local Gi* statistic was identified as space-time clusters by SaTScan. This is an important finding because it shows that simply targeting areas with high VL cases might not always be sufficient for intervention as the spatial and temporal aspects of the disease may portray a different scenario.

This study has several limitations. First, the findings of the study were based on populations who sought treatment at the respective UHCs; therefore, residents diagnosed outside of study area have not been included in the database. Second, we excluded 3680 cases whose addresses were outside the area of interest and only considered hospitalized cases. This would lead to some selection bias towards severe VL cases. But as opposed to outdoor patient information, this inpatient database had to have multiple scrutiny and had less chance of being a non-resident of our study area. Third, Skelly et al. [46] noted that passive surveillance data is prone to incorrect allocation of cases to geographic areas; we had no method for ascertaining this, so uncertainty about this issue is likely. Fourth, SaTScan searches for regular-shaped clusters, but in reality, true clusters can be irregular as well [47]. Finally, the study was based on human data only and could not integrate sand fly habitat data into the cluster analysis; therefore, the ecological influence on disease distribution could not be examined. As there is a severe lack of vector data in the study area, use of vector presence-absence data could provide a clearer picture of human-sand fly interaction and its contribution to the clustering of VL.

In spite of the limitations listed above, the main success of this study is the creation of a fine-scale regional map of VL hot spots and space-time clusters in highly endemic areas of the country. The lowest census tract was used in this work, which is administratively just above the household but below the subdistrict. Hence, the findings are easier to interpret as they are neither too detailed nor too general and can be used to directly assist policy makers in allocation of resources to VL endemic regions using a geographically constrained approach, such as considering one administrative unit (e.g., subdistrict) at a time, when formulating health policies [48]. Since complete elimination of VL from South Asia is highly unlikely [5], due to “one-size-fits-all” strategy, we believe the findings of this study could assist in the prevention of resurgence in existing areas or transmission to new areas.

## Conclusion

Of the numerous studies examining VL, very few have attempted simultaneous spatiotemporal analysis to understand the extent and degree of spatial heterogeneity. This is particularly true for Bangladesh, where VL is a considerable burden for impoverished communities, and detecting clusters can aid in targeted interventions. In this work, statistically significant clusters were identified through spatial analyses, and relative risks and likelihood ratios were calculated to compare VL occurrence within and outside the clusters, which could greatly assist public health officials to allocate proper resources at the strategic-planning level. In addition, these results could be highly useful for designing cost-effective strategies to control VL. Because global environmental change is expected to make new areas ecologically suitable for this fatal disease, the results of this work can help to identify exposed populations at greater risk so that transmission prevention strategies can be formulated. This study provided strong evidence of local clusters, which may be used to target vector control as destroying breeding sites or killing the sand fly vector have been found to be the most effective means of eliminating VL from South Asia [6]. Further, the identified clusters can assist in determining specific environmental and/or sociocultural properties associated with the distribution of VL. Doing so would help to reduce the disease burden in endemic areas of Bangladesh and elsewhere.

## Abbreviations

UHC: *Upazila* health complex
VL: Visceral leishmaniasis
